# Formation techniques for upper active channel in monolithic 3D integration: an overview

**DOI:** 10.1186/s40580-023-00411-4

**Published:** 2024-01-29

**Authors:** An Hoang-Thuy Nguyen, Manh-Cuong Nguyen, Anh-Duy Nguyen, Seung Joon Jeon, Noh-Hwal Park, Jeong-Hwan Lee, Rino Choi

**Affiliations:** 1 3D Convergence Center at Inha University, Incheon, 22212 South Korea; 2 Department of Materials Science and Engineering, Inha University, Incheon, 22212 South Korea

**Keywords:** Monolithic 3D, Thermal budget limitation, Upper layer, Techniques

## Abstract

The concept of three-dimensional stacking of device layers has attracted significant attention with the increasing difficulty in scaling down devices. Monolithic 3D (M3D) integration provides a notable benefit in achieving a higher connection density between upper and lower device layers than through-via-silicon. Nevertheless, the practical implementation of M3D integration into commercial production faces several technological challenges. Developing an upper active channel layer for device fabrication is the primary challenge in M3D integration. The difficulty arises from the thermal budget limitation for the upper channel process because a high thermal budget process may degrade the device layers below. This paper provides an overview of the potential technologies for forming active channel layers in the upper device layers of M3D integration, particularly for complementary metal-oxide-semiconductor devices and digital circuits. Techniques are for polysilicon, single crystal silicon, and alternative channels, which can solve the temperature issue for the top layer process.

## **Introduction**

Since the first integrated circuit was introduced, scaling down the device size in two dimensions (2D) has been the best method for increasing the integration density and device performance, resulting in increased profits in the semiconductor industry. On the other hand, 2D scaling has become increasingly difficult as the technology reaches smaller nodes, and the device size approaches those of molecules. Accordingly, attention has been turned to stacking semiconductor devices in three dimensions (3D) to maintain the progress of increasing device integration density predicted by Moore’s law. In 3D integration, there are two categories distinguished by the sequence of fabricating and stacking the lower and upper device layers. Monolithic 3D (M3D) integration, also known as sequential 3D integration, is a stacking process that involves fabricating a bottom layer device, followed by an active layer formed on top, and then a top layer for device fabrication. After these two device layers are prepared, they are connected by very small vias formed by the semiconductor photolithography process. On the other hand, in parallel 3D integration, two independent layers of devices are first fabricated separately. They are then stacked together at a later stage to achieve three-dimensional integration. To stack multiple device layers in parallel 3D integration, through-via-silicon (TVS) and bonding technology should involve vertical interconnects passing through a silicon wafer. Nevertheless, TVS and bonding technology have a limit to increasing the number of vertical interconnects. Wide diameters of TSVs are unavoidable because TSV and bonding technology have difficulties in producing long vias with high aspect ratios and aligning the dies, resulting in a small number of TSVs. Therefore, the other stacking method should be studied to utilize the ultimate benefits of 3D stacking. On the other hand, multi-layers of devices are processed step-by-step from the bottom to the top layer in the M3D integration process. The use of photolithography to make interlayer vias allows an extremely high interconnection density between the upper and lower device layers. Moreover, the delay time through shorter interconnection lengths (more than 10% of the total length) is shorter with a more cost-effective and single-flow process [1]. Thus far, studies have reported a high density of 2 × 10^7^ via/mm^2^ with the M3D process, which is approximately two orders of magnitude higher than TSV integration [2, 3]. A higher interconnection density provides higher bandwidth between the dies, which provides more freedom to design chips for advanced applications, such as the Internet of Things (IoT), artificial intelligence, and high bandwidth memory. This expanded bandwidth not only supports the creation of new devices but also allows for the integration of diverse technologies and materials within a single chip. This includes analog and digital components, micro-electro-mechanical systems, IoT sensors, or biomedical implants [4–6]. This technological convergence on a single chip paves the way for the development of more sophisticated and multifunctional devices and circuits, pushing the boundaries of what is achievable in semiconductor design and fabrication. Therefore, M3D technology has the potential for higher device density than TSV and bonding technology.

**Fig. 1:**
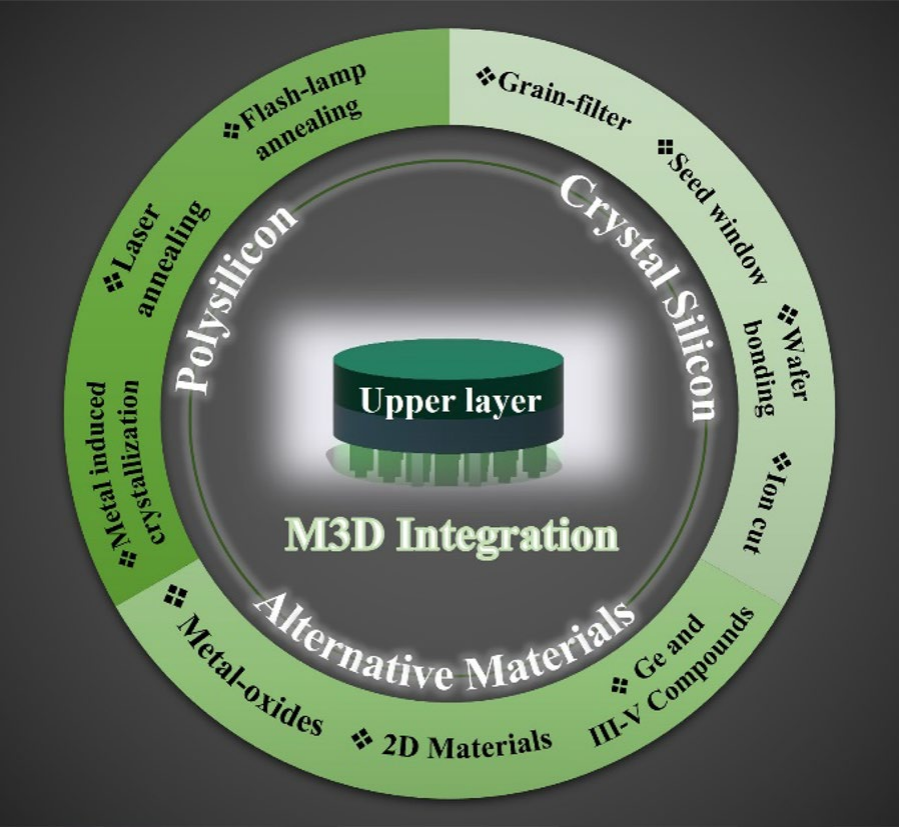
Techniques for high performances of top layers in the M3D structure

Despite the great benefits of M3D technology, it still faces several challenges in fabricating the upper channel, such as thermal budget, materials compatibility, process complexity, yield, and reliability. After completing the lower device layer covered with an interlayer dielectric, the upper device layer should be formed on top of this dielectric. The maximum temperature for the upper layer fabrication process should be lower than 500 ^o^C to protect the lower layer devices from high-temperature degradations, such as the deterioration of the metal wire, formation of silicide, and dopant diffusion [7]. In M3D integration, conventional front-end-of-line and back-end-of-line processes can be used to fabricate the lower device layer. On the other hand, the upper device layer should use materials compatible with the underlying layers, and fabrication processes should not induce additional defects or strain on the lower layer devices. They lead to the development of new processes, such as laser annealing to activate junction implantation, gate dielectric anneals, and silicide formation. In addition to device fabrication processes, M3D integration requires the formation of active channel material for upper device layers. This paper summarizes the techniques proposed for forming upper channels in M3D integration that require low processed temperatures. They can be categorized by channel materials, such as polycrystalline silicon (poly-Si), single-crystal silicon (SCS), and alternative channel materials, as shown in Fig. 1. Each technology has advantages and limitations, and researchers have examined new and innovative approaches to overcome the challenges associated with fabricating upper channels in M3D.

## Polycrystalline silicon

### Deposition of amorphous silicon and crystallization into poly-Si

Poly-Si is comprised of multiple small silicon grains with different crystallographic orientations. Although the carrier mobility of poly-Si is relatively lower than that of single crystalline Si, it is significantly higher than amorphous Si (50−100 cm^2^V^-1^s^-1^ and 1 cm^2^V^-1^s^-1^) [8]. Poly-Si should have a high degree of crystallinity and low levels of impurities and defects to achieve good performance. The grain size of poly-Si is also important because larger grains generally result in higher carrier mobility. Furthermore, the surface of poly-Si should be smooth to reduce surface scattering and enhance carrier mobility. Recent advances in poly-Si channel engineering focus on low-temperature processes, strain engineering, and advanced gate stack engineering to enhance transistor performance. Innovation Gate-All-Around transistors, alternative channel materials, quantum dot transistors, and effective process integration contribute to improved scalability and performance. Ongoing developments aim to address challenges and enable the design of more efficient semiconductor devices.

Amorphous Si can be deposited at low temperatures using various techniques, such as low-pressure chemical vapor deposition (LPCVD), plasma-enhanced chemical vapor deposition (PECVD), and sputtering. In PECVD, the deposition temperature can be as low as 300−400 ^o^C. This technique uses plasma to break down the precursor gases into reactive species that deposit on the substrate. Similarly, sputtering can also be performed at relatively low temperatures, around 200−400 ^o^C, depending on the specific process parameters. Various techniques have been applied to recrystallize the amorphous phase to poly-Si, such as laser annealing (LA), metal-induced crystallization (MIC), metal-induced lateral crystallization (MILC), and flash-lamp annealing (FLA).

### Laser annealing

**Fig. 2:**
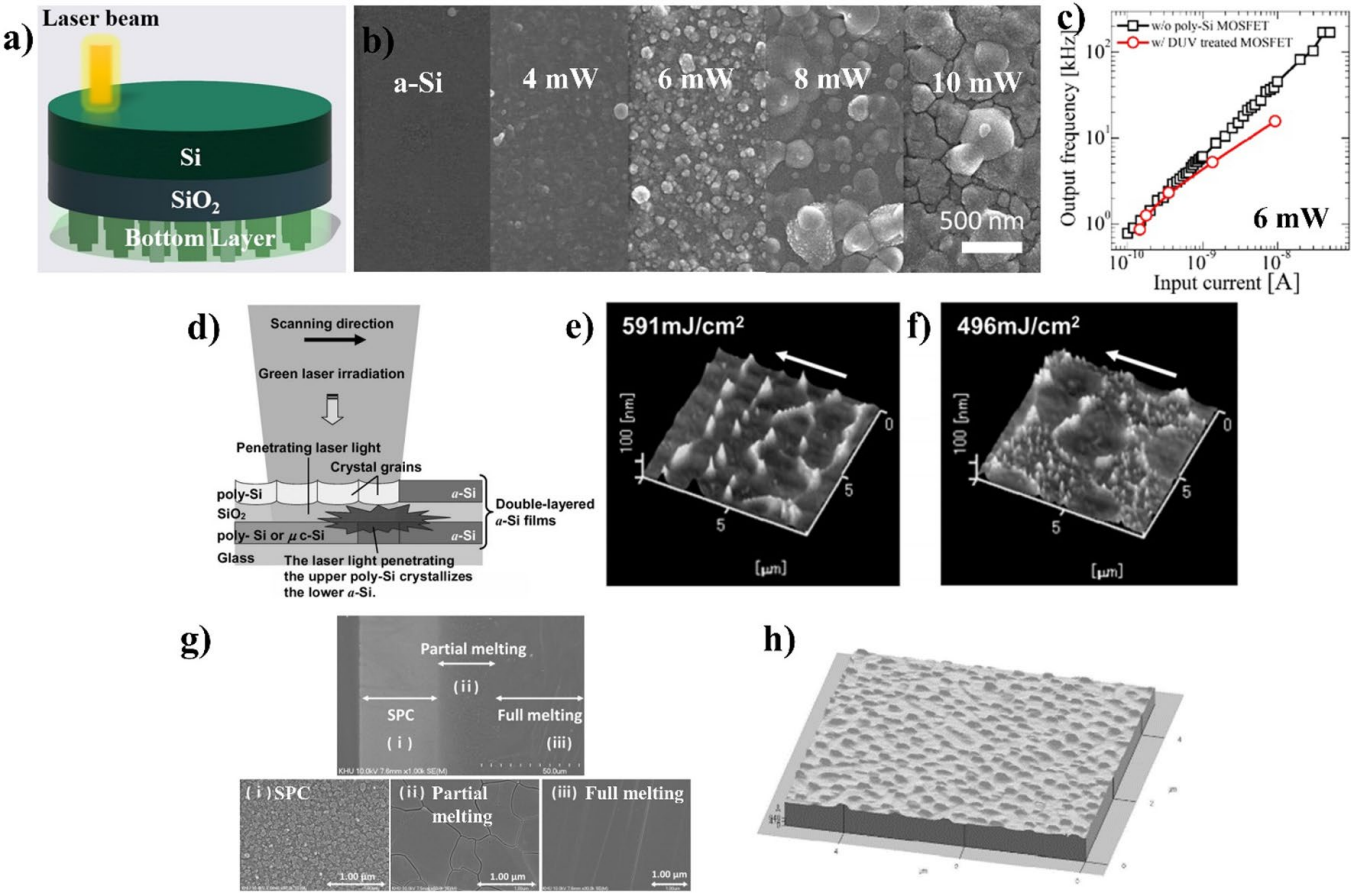
**a** Laser annealing process for the M3D structure **b** SEM images for the a-Si surface are annealed at various DUV laser power of 4, 6, 8, and 10 mW **c** the degradation of the bottom device after LA at 6 mW compared to without laser treatment [9] **d** Green laser annealing for double a-Si layer and **e** surface of single-layer poly-Si compared to **f** upper layer in the double poly-Si layer structure [10] **g** Formed polysilicon film by a single blue laser scan with various areas due to Gaussian profile of laser beam [11] **h** Hillocks appear on the edge of the grain boundary of the polysilicon after LA from the AFM result [12]

LA is a powerful technique to recrystallize amorphous Si into polysilicon in M3D integration. This technique has already been used to manufacture the backplane of flat panel displays for low-temperature polycrystalline silicon (LTPS). The process involves the absorption of laser energy by amorphous silicon, increasing the temperature to exceed its melting point. This results in the melting and recrystallization of amorphous silicon into large grain-size polysilicon. Laser energy causes the movement of the atoms in amorphous silicon, leading to the formation of a crystalline structure with higher carrier mobility poly-Si. Achieving high-quality poly-Si during laser annealing involves carefully controlling temperature. It is essential to maintain the temperature within the a-Si layer above its melting point (1420 K) for effective transformation while ensuring it stays below the boiling point of silicon (3538 K) to prevent vaporization. Simultaneously, the temperature of the SiO_2_ layer beneath the a-Si layer must be kept below the melting point of SiO_2_ (1986 K) to prevent thermal damage [13]. This meticulous temperature control is critical for optimizing the crystallization process and preserving the integrity of the underlying SiO_2_ layer [14]. Several presented laser types have recently been demonstrated for solid-phase crystallization of amorphous Si or activating the doping channels, such as excimer lasers, solid-state lasers, continuous wave (CW) lasers, and pulsed lasers with various wavelengths [15–17]. The choice of laser type depends on the specific requirements of the application, such as the desired temperature, annealing time, and spatial resolution. Most lasers do not have a sufficiently large field size to cover an entire die at once owing to economic reasons. Consequently, the laser beam must be scanned when laser annealing is performed. The annealed area is divided into three sections: central region, transition region, and edge region. Thus, it is crucial to carefully evaluate the kinetics of melting and recrystallization at the edge of the laser to ensure complete crystallization of the entire area.

Among various laser types, shorter wavelength lasers, such as ultraviolet (UV) or deep ultraviolet (DUV) excimer lasers with wavelengths of 308 nm and 193 nm, respectively, are widely operated for reaching good performance because of the high laser power and large absorption coefficient of a-Si [9, 18, 19]. UV excimer lasers are advantageous because they are strongly absorbed by silicon. In addition, a larger beam with a higher energy density than other laser light sources is available [20]. The other advantage of UV lasers is that the high photon energy of UV laser enables the fabrication of the upper active layer without damaging the devices on the bottom layers because the heat is confined in a very localized area. Nano second UV-LA can also be used in crystalline poly-Si gates and active devices to reduce the chip size and power consumption [7, 21]. In the experiment with DUV (266 nm for wavelength), the annealing time of all samples with different annealing powers was fixed at 25 ms, as shown in Fig. 2b. At a laser power of 8 mW, the sheet resistance of Si for driving the transistor on the top layer was obtained by rapid thermal annealing. In addition, the grain size of a few hundred nanometers was achieved at a laser power of 8 and 10 mW. The high performance of the current-to-frequency ring oscillator on the bottom layer suggests the least degradation of the bottom complementary metal-oxide-semiconductor (CMOS) in case of 6 mW annealing for greatest mobility as in Fig. 2c. Hence, DUV is one of the effective solutions for M3D [9].

Recently, other wavelengths have been evaluated for a-Si crystallization, such as green laser annealing and blue laser diode annealing [10, 11, 16, 17, 22, 23]. For CW green laser crystallization, the production costs can be reduced, and laser power stability, larger polycrystalline grains, and higher carrier mobility can be achieved. On the other hand, high power and thick a-Si films are required because of the low optical absorption coefficient of a-Si. The green laser has a lower absorption coefficient in poly-Si than the excimer laser, meaning that most of the laser energy passes through the Si thin film. Y. Sugawara et al. used double-layered Si thin-film substrates consisting of two a-Si layers and a SiO_2_ interlayer to overcome this problem, as shown in Fig. 2d. By annealing and crystallizing the upper a-Si layer using green laser irradiation, the lower a-Si layer absorbs the green laser light passing through the poly-Si and crystallizes. The heat from the upper layer reduces the thermal gradient in the vicinity of the melt, leading to an extension of its melting times and an increase in its grain size (~ 2 μm) shown in Fig. 2e–f. Essentially, the lower a-Si is believed to act as a heat reservoir during crystallization.

Semiconductor blue-multi-laser-diode annealing (BLDA) has a greater penetration depth compared to UV lasers (532 and 308 nm), and it demands a lower threshold energy density for crystallizing near-surface amorphous silicon compared to the green or near-infrared alternatives. Figure 2g) presents three different growth regions depending on the energy and temperature distribution during a single scan, showing the crystallization of varying grain sizes ranging from 50 to 200 nm [11, 22]. In terms of thin-film transistor (TFT) performances, S. Jin et al. established the uniformity and device quality in electrical properties across different thicknesses (75, 100, and 125 nm) through BLDA activation [11]. The highest mobility of 134 cm^2^V^-1^s^-1^ and the largest on-off ratio of 10^8^ were achieved with 125 nm of thickness. BLDA covers a wide range of thicknesses and grain sizes effectively. Furthermore, BLDA has been explored for its high stability and cost-efficient, low-installation attributes.

Despite the significant benefits of LA, the regrown Si and surface tension produce hillocks and increasing roughness, as shown in Fig. 2h [7, 12]. One potential solution is the application of a thick dielectric cap on the top of amorphous silicon before the LA process. On the other hand, this approach introduces the formation of wrinkles, which increases with the depth of melted Si [24, 25]. Therefore, careful consideration during the design phase of laser annealing processes is essential to mitigate this phenomenon.

### Flash-lamp annealing

**Fig. 3:**
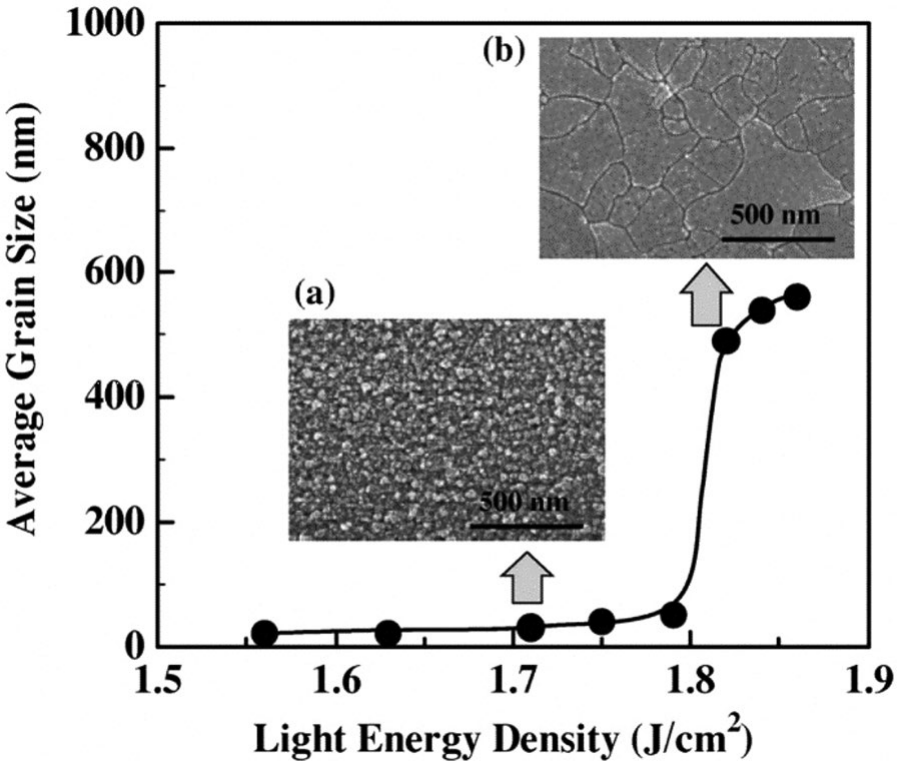
Average grain size is controlled by light energy density from 1.56 to 1.82 J/cm^2^ [26]. When subjected to thermal energy below 1.8 J/cm^2^, the a-Si thin film undergoes incomplete melting, resulting in the formation of small-grained poly-Si **a** as opposed to the development of larger grains **b** observed at higher thermal energy levels

FLA utilizes an array of xenon flash lamps to generate intense pulsed light, rapidly heating the material surface. This rapid heating leads to exceedingly short annealing times, typically from microseconds to milliseconds. This technique can produce high-quality poly-Si with large grains. This technique has considerable advantages over the LA technique, such as high heating and cooling rate, large area coverage, uniformity, and cost-effectiveness [26, 27]. F. Terai et al. achieved a grain size of 500 nm at a light energy density of 1.82 J/cm^2^ during Xe FLA without the necessity of substrate heating shown in Fig. 3. The light is completely absorbed before a depth of 50 nm because of the slight differences in light absorption energy between the surface and inner layer of the a-Si film. Once the light energy exceeds a threshold, the entire a-Si film melts simultaneously, resulting in the growth of large-grain poly-Si caused by crystallization from both the surface and inner layers. On the other hand, FLA also has some limitations. A critical hurdle in FLA lies in achieving consistent temperatures across the entire wafer, vital for uniform crystal quality and electrical performance. Precise control of the lamp pulse, managing both duration and intensity, is pivotal in realizing this uniform heating. To alleviate thermal stress on the wafer and the associated risks like cracking, a preheating system is employed, reducing temperature gradients between the front and back sides [28]. Moreover, safeguarding devices in lower layers from thermal damage involves the strategic use of additional insulating layers or materials, acting as a protective barrier against excessive heat transfer. Additional measures must be taken to ensure high reproducibility and homogeneity [29].

### Metal-induced crystallization and metal-induced lateral crystallization

MIC and MILC are techniques to produce large-grain polycrystalline silicon films on non-crystalline substrates, such as glass. These techniques are based on metal-induced growth, which involves using metal as a catalyst to induce the growth of Si crystals.

**Fig. 4:**
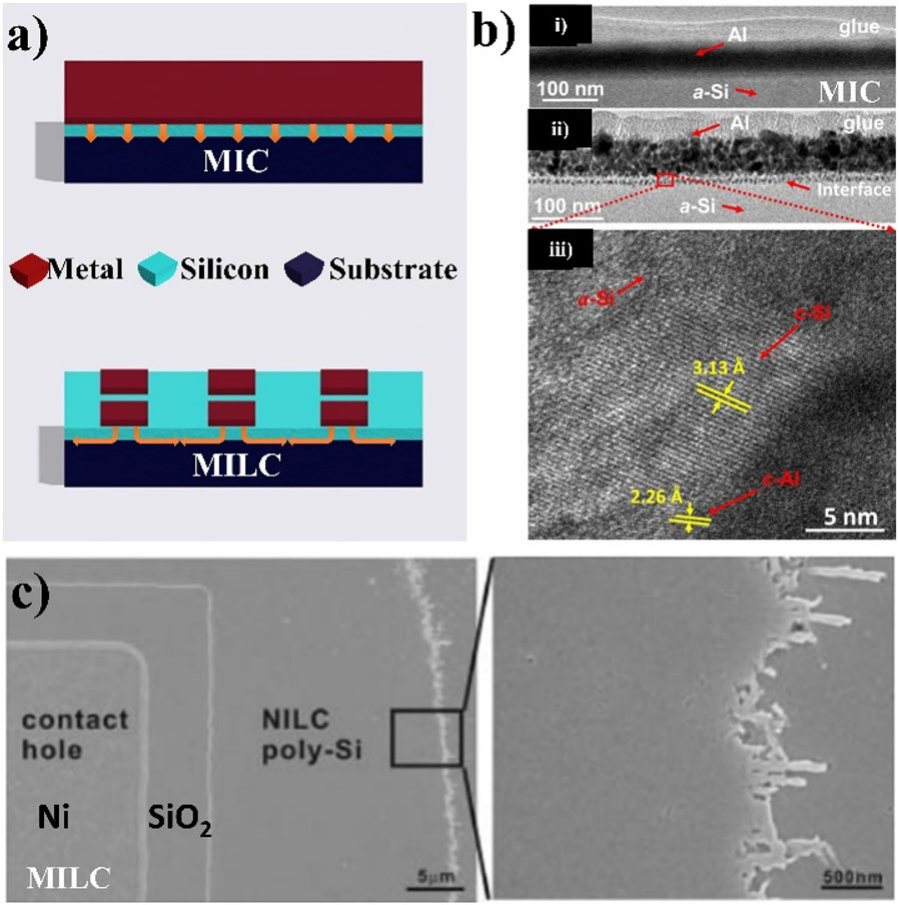
**a** Metal-induced crystallization and metal-induced lateral crystallization process **b** a-Si crystallizes as poly-Si under the Al layer in the MIC process, at which the annealing temperature is 200 ^o^C [30] **c** Poly-Si is lateral crystallized from the Ni deposited in the contact hole and annealed at 530 ^o^C for 15 h to form the NILC poly-Si [31]

MIC involves the deposition of a thin layer of metal (such as Ni or Al) on top of the a-Si film, followed by high-temperature annealing of the metal/a-Si film as in Fig. 4a [32–34]. The metals used in MIC are categorized into two groups based on their crystallization mechanisms: eutectic-forming metals and silicide-forming metals. Eutectic metals (Al, Ag, or Au) induce poly-Si formation by exchanging adjacent Si and metal films, which occurs during the transition from amorphous to polycrystalline. In the case of silicide-forming metals (Ni or Pd), the metal layer catalyzes the crystallization of the a-Si film, forming a thin layer of metal silicide at the metal and Si interface during annealing. The metal silicide layer acts as a nucleation site for the growth of large, parallel silicon grains that extend laterally across the surface of the a-Si layer. As the annealing progresses, these silicon grains grow and coalesce, resulting in a highly crystalline polycrystalline silicon film with large grains. The correct location of crystallization and reduction in the required temperature is managed by controlling the balance of changes in the interface energy and semiconductor energy. The process is simple and can be performed at relatively low temperatures (< 550 ^o^C), making it a cost-effective method for producing poly-Si films. The primary mechanism of MIC can be divided into four parts, including the weakening of covalent bonds, wetting of the grain boundaries, exchange of layers, and nucleation and growth [35].

The microstructure and growth kinetics in the MIC process depend on various important parameters. The effects of the thermal budget (temperature and time), types of metal, the thickness ratio of metal to semiconductor, and substrates have been examined and discussed. The nucleation rate decreases as the annealing temperature is lowered, leading to a larger grain size. At lower temperatures, diffusion is difficult, which may cause the development of larger depletion regions around the growing grains. The growth of existing grains before they impinge inhibits new nucleation. Therefore, the crystallization process takes longer at lower annealing temperatures [36]. For choosing the types of metal, the post-transition metal, such as Al shown in Fig. 4b, can reduce the MIC temperature without forming a compound phase in the MIC process (about 200 °C), while transition metals, such as Ni and Pd, form the multiple compound phase with Si [35]. The a-Si layer should be thicker than the Al layer based on some studies to prevent the porous poly-Si layer [37, 38].

MILC is a variant of MIC where the metal layer (Ni, Co, or Pd) is patterned into a series of parallel strips or lines on top of the a-Si film, as shown in Fig. 4a, c [31, 39–41]. The metal lines melt and induce lateral crystallization of the a-Si film perpendicular to the metal lines during the annealing step. As a result, highly aligned poly-Si grains form along the metal lines, resulting in a highly ordered poly-Si film. The MILC process is scalable and capable of producing large-area poly-Si films with favorable electrical properties.

Compared to MIC, MILC has several advantages, such as higher grain size and improved electrical properties because of the lateral growth of the poly-Si grains. Furthermore, metal patterning allows for greater control over the orientation and alignment of the poly-Si grains. The process, however, is more complex and requires additional steps for metal patterning and alignment. In addition, the process temperature of MILC is higher than MIC. Although a lower annealing temperature of approximately 450 ^o^C for Ni MILC has been achieved, further investigation is necessary to achieve even lower process temperatures [39].

Both MIC and MILC are low-temperature processes capable of producing extensive poly-Si areas with good crystal quality and uniformity. These techniques require neither expensive equipment nor complex processing steps. Nevertheless, potential metal diffusion into the a-Si film is a drawback, leading to contamination. The eutectic phase forming metals reduces the crystallization temperature and forms a large grain size of 10 μm, but the intermixing of metal atoms with the silicon lattice leads to the degradation of the performance and reliability of the devices. On the other hand, the silicide phase forming metals can enhance the mobility and the transfer characteristics of the devices by reducing the contact resistance of gate and source/drain regions with some unique silicide phases, such as nickel monosilicide. Precise control of the amount of diffused metal atoms and the annealing temperature is crucial to prevent adverse effects on the resulting devices.

## Single-crystal Si

Obtaining high-quality silicon channels for the upper device layer through a low-temperature process is a primary challenge in M3D integration. Single-crystal silicon is always preferable to poly-Si because it offers superior performance, such as high mobility and reliability, and circumvents the formation of defects. Among the various techniques to grow SCS for the upper active channels, epi growth of Si through seed windows, the µ-Czochralski (grain-filter) process, and wafer bonding techniques have been introduced.

### Seed window

The seed window technique involves melting amorphous Si films and crystallizing them from single-crystal Si seeds grown from the Si substrate through contact holes. This has been achieved through selective epitaxial Si deposition, producing perfect SCS films on the oxide layer.

**Fig. 5:**
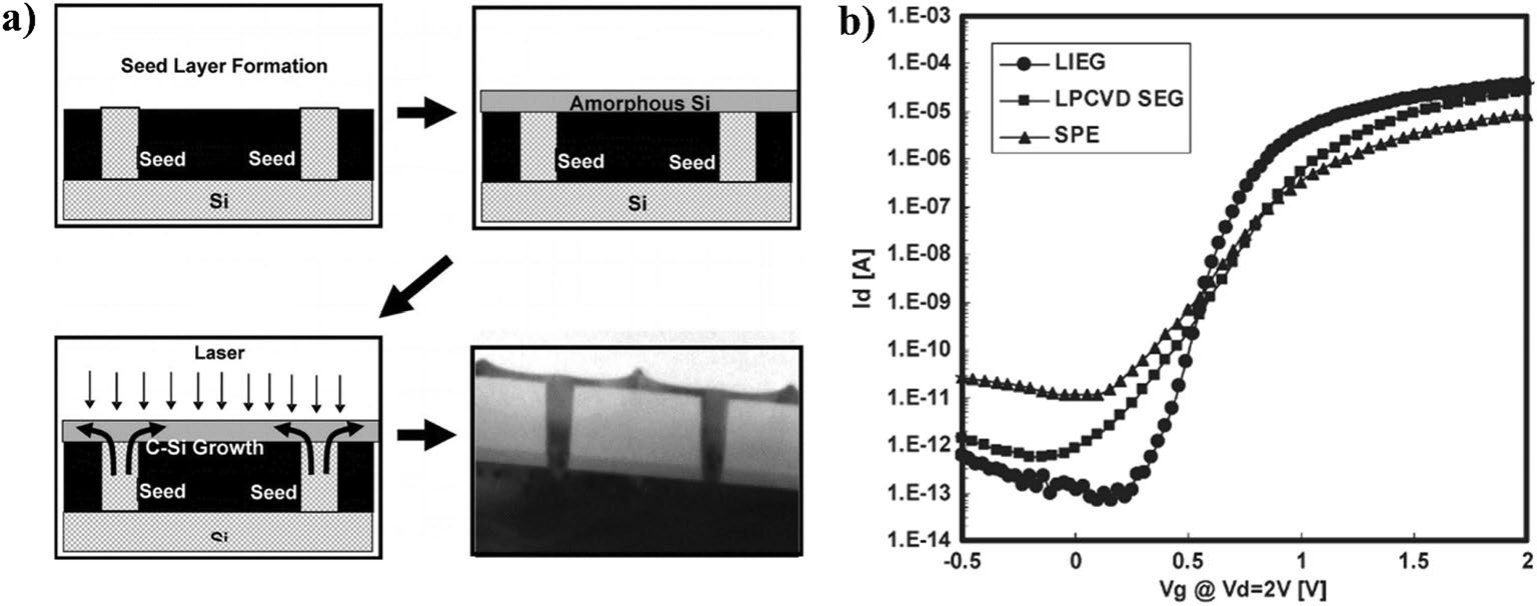
**a** Seed window process for laser-induced SCS [42] **b** Comparison of the electrical performance of various crystallization techniques as laser-induced epitaxial growth (LIEG), LPCVD selective epitaxial growth (LPCVD SEG) at 800 ^o^C, and furnace-annealed sold phase epitaxy (SPE) at 580 ^o^C [42]

The standard process for the seed window technique involves patterned contact holes through an interlayer dielectric (ILD) that connects the underlying silicon substrate with the stacked layer, as shown in Fig. 5a. Subsequently, the seed is grown through the contact holes by conventional selective epitaxial growth from a single crystalline substrate. The surface is flattened thoroughly by chemical-mechanical polishing (CMP) to eliminate facets and smooth the topology. LPCVD a-Si film deposition is followed to cover the seed and ILD layers. UV laser annealing, green laser annealing, or spike rapid thermal annealing induce the formation of single-crystalline structures [42–44]. The region affected by laser annealing can be classified into three segments owing to its Gaussian profile: partial melting, near-complete melting, and complete melting. Within the near-complete melting range, solid phase islands remain after melting, and super lateral growth can begin from these islands before merging with adjacent grains, resulting in larger grains. This lateral epitaxial growth process is a controlled super lateral growth phenomenon, where growth occurs laterally from seeds. The heat produced during laser irradiation may escape through the contact hole filled with SCS because Si has a higher thermal conductivity than oxide. As a result, the molten silicon solidifies from the top of the contact hole, which serves as a seed for epitaxial vertical and lateral growth.

The I-V curves of NMOS TFTs fabricated using various techniques such as laser-induced epitaxial growth (LIEG), LPCVD selective epitaxial growth (LPCVD SEG) at 800 ^o^C, and furnace-annealed sold phase epitaxy (SPE) at 580 ^o^C were compared to evaluate the quality of SCS by seed window technique, as shown in Fig. 5b. The green laser annealing with the seed window process shows the best electrical performance resulting from the best crystal quality of the channel Si [42].

### µ-Czochralski (grain-filter) process

In addition to the seed window process, the µ-Czochralski (grain-filter) process is also one of the promising candidates for forming high-quality SCS as the grain location-control technique.

**Fig. 6:**
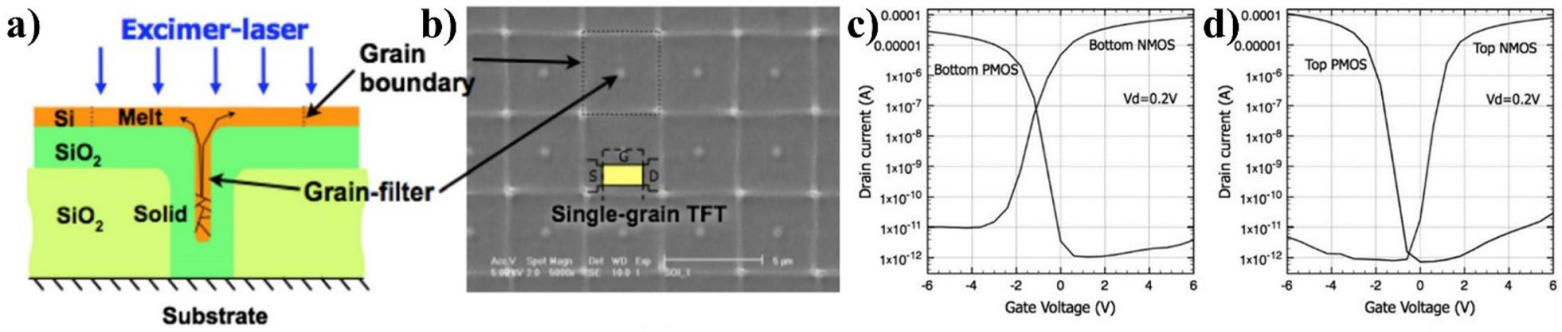
**a** Grain-filter process with UV laser annealing for SCS and **b** single-grain TFT. Comparison of the electrical performance of n- and p- MOS and SCS TFTs at **c** bottom and **d** top layers with the grain-filter process for top TFTs [45]

R. Ishihara et al. examined the M3D integration with SCS for the upper channel by the grain filter process, as shown in Fig. 6a [45]. The grain filter was patterned on SiO_2_ with a diameter and a depth of 100 and 700 nm. A 250 nm a-Si layer was deposited on the SiO_2_ layer by LPCVD at 550 ^o^C. A single excimer laser pulse was radiated on the heated substrate (450 ^o^C) for the crystallization process. When high energy densities are used, lateral grain growth occurs through a vertical growth phase through a narrow hole. Typically, grains become occluded during the vertical regrowth of partially molten silicon, decreasing the number of grains growing. Consequently, if several seeds are present in the original unmolten part, some will be occluded during this vertical growth phase [46]. This process enhances the yield of monocrystalline islands. After annealing, Si grains, 6 μm in diameter, were grown on the positions of the grain filter, as shown in Fig. 6b.

The electrical performance of the bottom and top devices was compared to assess the quality of SCS crystallized by the grain-filter process, as shown in Fig. 6c, d. The TFTs fabricated on both layers exhibit comparable transfer characteristics, indicating the good crystal quality of the top Si channel. High mobilities were extracted from the I-V curves of 600 and 200 cm^2^V^-1^s^-1^ for top-nMOS and pMOS. The other study also reported the outstanding performance of TFTs on silicon channels crystalized by the grain-filter process with high mobility of 430 cm^2^V^-1^s^-1^ and small SS of 0.39 V/dec [47]. Despite precise control of the location of each grain by the position of the grain filter in this process, the control of grain orientation is a substantial challenge in achieving uniform device fabrication. The primary issue is the grain misorientation or nonuniformity across the entire wafer. Despite careful control of the growth parameters, such as growth conditions or laser parameters, achieving a uniform alignment and orientation of the grains is difficult. Variations in crystallographic orientation can lead to changes in electrical performance and reliability.

### Wafer bonding

**Fig. 7:**
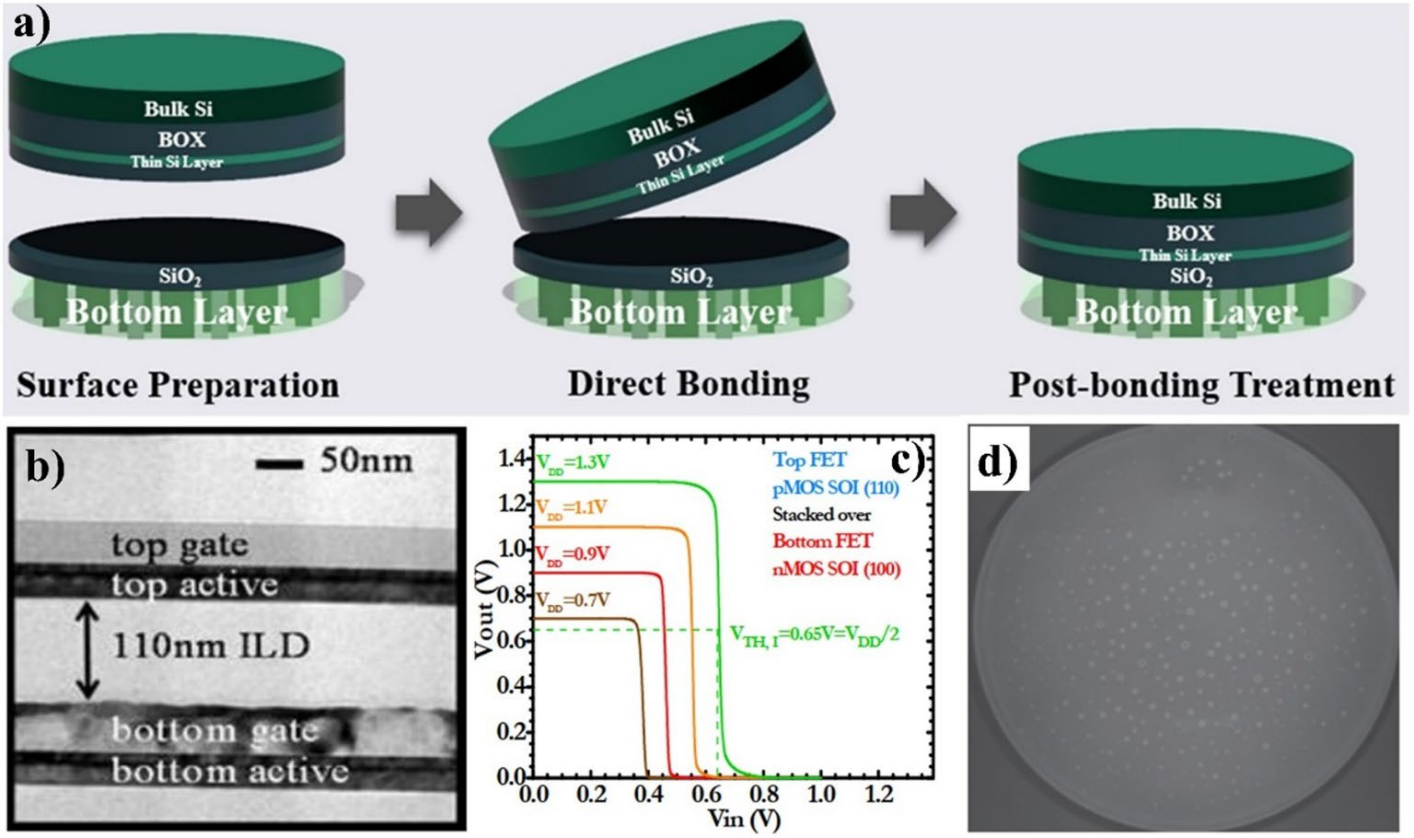
**a** Direct wafer bonding between two insulator surfaces **b** Top and bottom CMOS layers by the wafer bonding process and **c** transfer voltage performance of an inverter with the top pFET and bottom nFET [52] **d** voids appear after heat treatment SiO_2_ bonding [50]

Wafer bonding of a single crystal Si layer on the bottom device wafer is the most popular process in forming an upper active layer in the 3D structure of integration. This approach is stable, straightforward, easily implementable, and suitable for various applications, all achieved at low temperatures. The basic process of this technique encompasses surface preparation, direct contact, adhesion, and bonding across two clean and flat surfaces without an intermediate layer, as shown in Fig. 7a [48]. Highly flat and no defect surfaces are essential for the wafer bonding process for high-yield and strong bonding. Parameters, such as bonding energy, surface cleanliness, roughness, and flatness play a pivotal role in achieving high-quality bonding. The surface of the bonded wafers can be prepared using standard cleaning processes, such as RCA1 or SC1 (NH_4_OH: H_2_O_2_: H_2_O = 1:1:5) and RC2 or SC2 (HCl: H_2_O_2_: H_2_O = 1:1:6), to remove all organic compounds and ionic contamination [49, 50]. In addition to the wet cleaning process, the dry surface preparation processes, such as plasma activation or UV/O_3_ process, also result in high-quality bonding. For roughness, the root mean square of less than 1 nm is required for hydrogen bonding at room temperature [51]. The roughness can be controlled by the CMP process. Finally, the flatness of the bonded surfaces is vital for preventing unbounded areas. The bonding mechanism between two surfaces is achieved through either van der Waals (vdW) forces or hydrogen bonding. Owing to the relatively moderate strength, annealing at elevated temperatures is often required after room temperature bonding to fortify the bonding strength. Plasma-activated or other techniques can reduce the annealing temperature for M3D integration.

Based on the wafer bonding theory, the CEA Leti group reported the feasibility of M3D CMOS integration for 22 nm technology nodes, with the upper layer fabrication subjected to a maximum temperature of 600 ^o^C, as shown in Fig. 7b, c [52]. They achieved this by bonding an SOI substrate for the upper active layer at a low temperature of 200 ^o^C. No degradation in the performance of the bottom devices was observed, highlighting the effective management of the heat budget during the fabrication of the top layer. Encouraged by these initial results, the group developed an industrial-scale process for full M3D CMOS over CMOS CoolCube^™^ on a 300 mm wafer [53]. Their research employed a direct bonding technique to transfer a 10 nm Si layer with a bonding annealing temperature of 300 ^o^C. Although the maximum temperature for the upper device fabrication process reached approximately 650 ^o^C, they successfully reduced the highest thermal budget through low-temperature epitaxy and low-k spacers, ensuring it remained below 550 ^o^C. The group achieved high alignment precision and low defect density across the entire wafer, further validating the robustness of their process.

Wafer bonding in M3D can integrate similar materials, such as silicon and III−V compounds, with different thermal expansion coefficients and lattice constants. On the other hand, while wafer bonding offers these benefits, it can introduce contamination from the transfer materials or environment during the transfer process and can degrade the purity of the silicon surface. Contaminants may introduce unwanted impurities that affect the performance of the integrated device. For example, after heat treatment, voids were formed from the –OH group reaction between two surfaces, as shown in Fig. 7d [54]. The transfer process can introduce stress and strain to the silicon layer. Managing these mechanical forces is crucial to prevent deformation or cracking, which could impair the performance of the transferred layer. Large-size wafer bonding requires careful management of bowing, warpage, and microroughness to ensure uniformity across a large area. The high cost of SOI is a critical drawback on the other side of direct wafer bonding.

### Ion cut

**Fig. 8:**
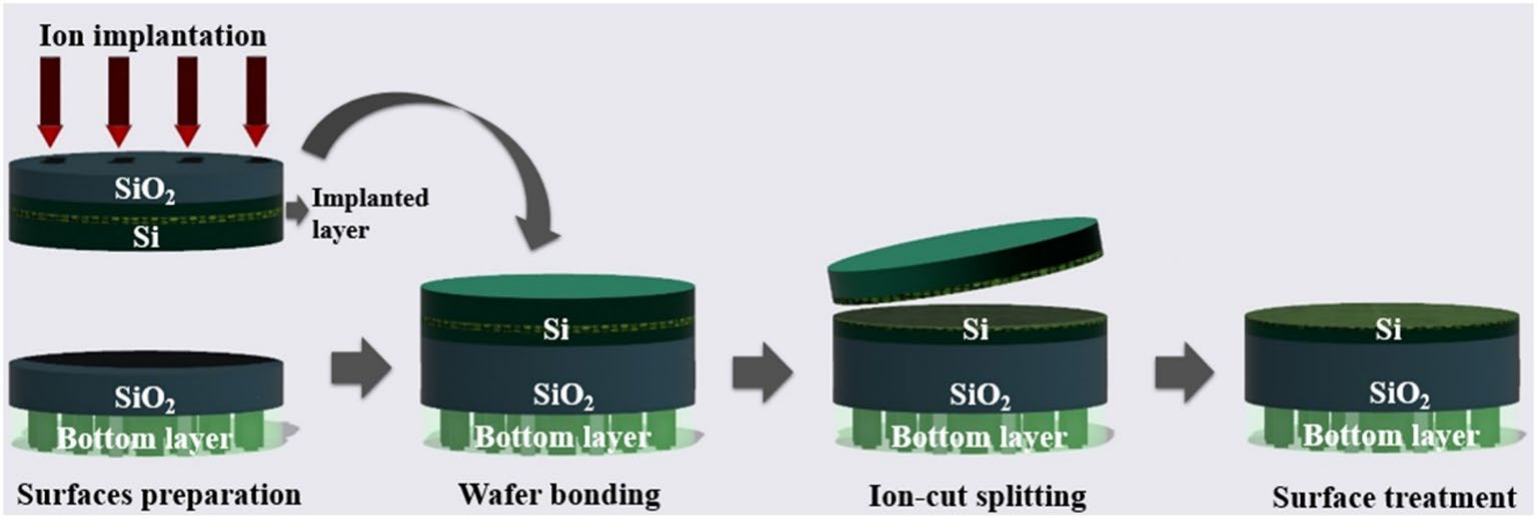
Basic ion-cut process for bonding the upper channel layer

**Fig. 9:**
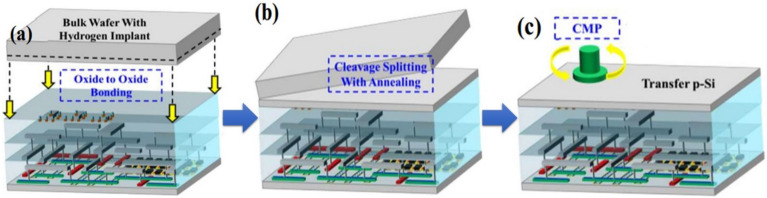
**a** Ion-cut and bonding process for M3D structure **b** The maximum temperature for the cleavage process is under 500 ^o^C **c** The surface of transfer Si is treated by post-CMP. Figure reproduced from ref. [55]

Ion-cut is a process that separates a thin layer of semiconductor material from the bulk materials transferred and stacked on top of another layer, as shown in Fig. 8. In this method, a high dose of hydrogen ions is implanted. A hydrogen ion layer is formed, which becomes a buried layer of microbubbles by various doping doses. This layer is easy to cleave under the annealing process due to the expansion of microbubbles and releases thin Si film, as shown in Fig. 9 [55]. In the other study, a thin Si layer is bonded using a bonding and de-bonding process at a temperature below 250 ^o^C [56]. Ion-cut is compatible with a wide range of semiconductor materials, including silicon, germanium, III−V compounds, and silicon carbide [57–59]. On the other hand, the ion-cut process also has some limitations, such as the difficulty of achieving high process yields and controlling the ion dose and energy for microbubble layers.

## Alternative channels

One of the major advantages of M3D integration is integrating different materials on top of high-performance Si device layers. Various alternative materials have been assessed as an alternative to silicon as the upper channels. Metal oxides exhibit high electron mobility, are transparent, and flexible, making them suitable for TFTs in display technologies. 2D materials offer unique electronic properties promising enhanced performance in M3D circuits. Ge and III-V compounds, with their higher electron and hole mobility compared to silicon, can contribute to faster transistor switching speeds. These alternative materials address specific limitations of silicon, such as thermal issues and scaling challenges, while also enabling the design of novel devices and circuits.

### Metal oxides

**Fig. 10:**
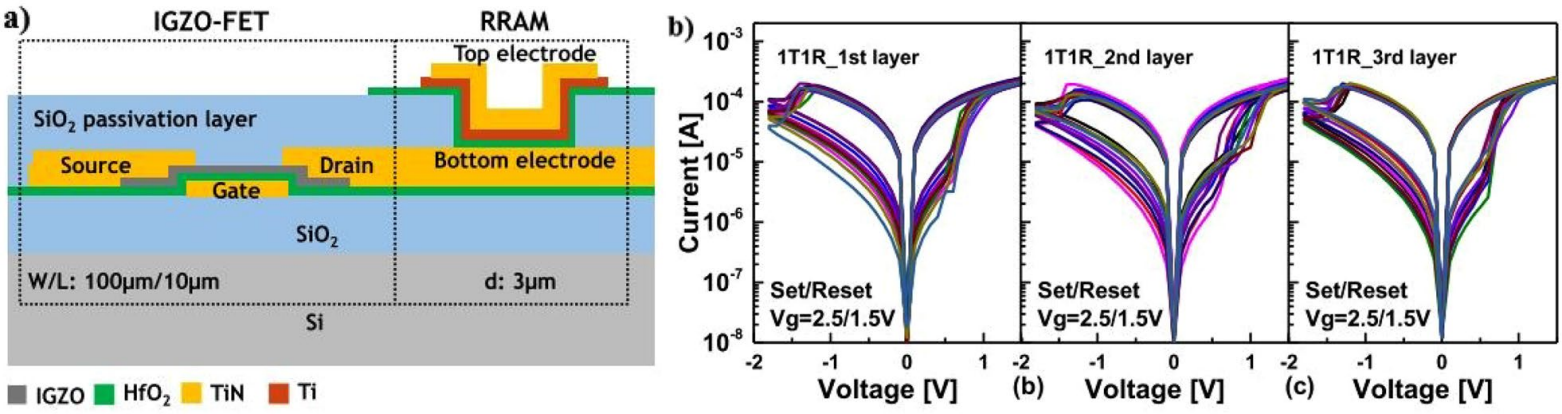
**a** IGZO TFT in 1T1R RRAM structure and **b** electrical performance of three layers of RRAM [60]

Metal oxides, such as indium-gallium-zinc-oxide (IGZO), indium-zinc oxide (IZO), zinc oxide (ZnO), indium oxide (In_2_O_3_), copper oxide (CuO), and tin oxide (SnO), are attractive for thin-film transistor applications. Their high mobility, transparency, and compatibility with large-area and flexible substrates give them applications in displays, sensors, and other electronic devices. Among the various oxide semiconductors, IGZO is an oxide semiconductor material that has attracted attention for its potential applications in M3D integration. IGZO has several desirable properties, including higher mobility, smaller subthreshold swing, and better stability. These properties make it a promising candidate for use as an active layer in the upper channels of M3D. Several studies have reported the successful deposition of IGZO on SiO_2_ substrates using various techniques, including sputtering and atomic layer deposition. Radio-frequency sputtering is commonly employed to deposit IGZO thin films at room temperature, followed by annealing to obtain good electrical performance. The maximum temperature for the whole process should be lower than 400 ^o^C to avoid the degradation of the bottom devices. The IGZO TFT for resistor random access memory (RRAM) array was successfully demonstrated for the M3D structure in combination with RRAM for the 1T1R device, as shown in Fig. 10 [60]. From a single device, the array is built based on IGZO TFT at 400 ^o^C of a limited temperature without the degradation of differential devices. In recent developments, there has been a surge in research focused on achieving low-temperature fabrication, creating thinner channels (about 10 nm), and enhancing the quality of thin films through the ALD process. This exploration encompasses a range of oxide semiconductors, including but not limited to In_2_O_3_, Ga-Sn-O, and In-Ga-Sn-O [61, 62]. These advancements hold promising prospects for the successful implementation of the M3D process.

In contrast to n-type metal oxide semiconductors, such as IGZO and IZO, which exhibit excellent mobility (~ 100 cm^2^V^-1^s^-1^) and stability, achieving high-performance p-type metal oxides presents significant challenges. P-type materials face obstacles, such as a narrow fabrication window, sensitivity to temperature, and poor electrical characteristics, including low mobility, low on/off current ratio, and large subthreshold swing. Despite many p-type materials, such as SnO, Cu_2_O, and NiO, and efforts to enhance the performance, they have not achieved the performance levels of their n-type counterparts. This performance gap between n-type and p-type materials imposes limitations in realizing CMOS technology in upper device layers. In the case of combining with a Si channel device, although the IGZO has good electrical performance, it still has a low frequency. Several efforts have been made to ameliorate using a crystalline IGZO channel or Ta_2_O_5_ gate dielectric, but it is incompatible with the M3D process.

### 2D materials

**Fig. 11:**
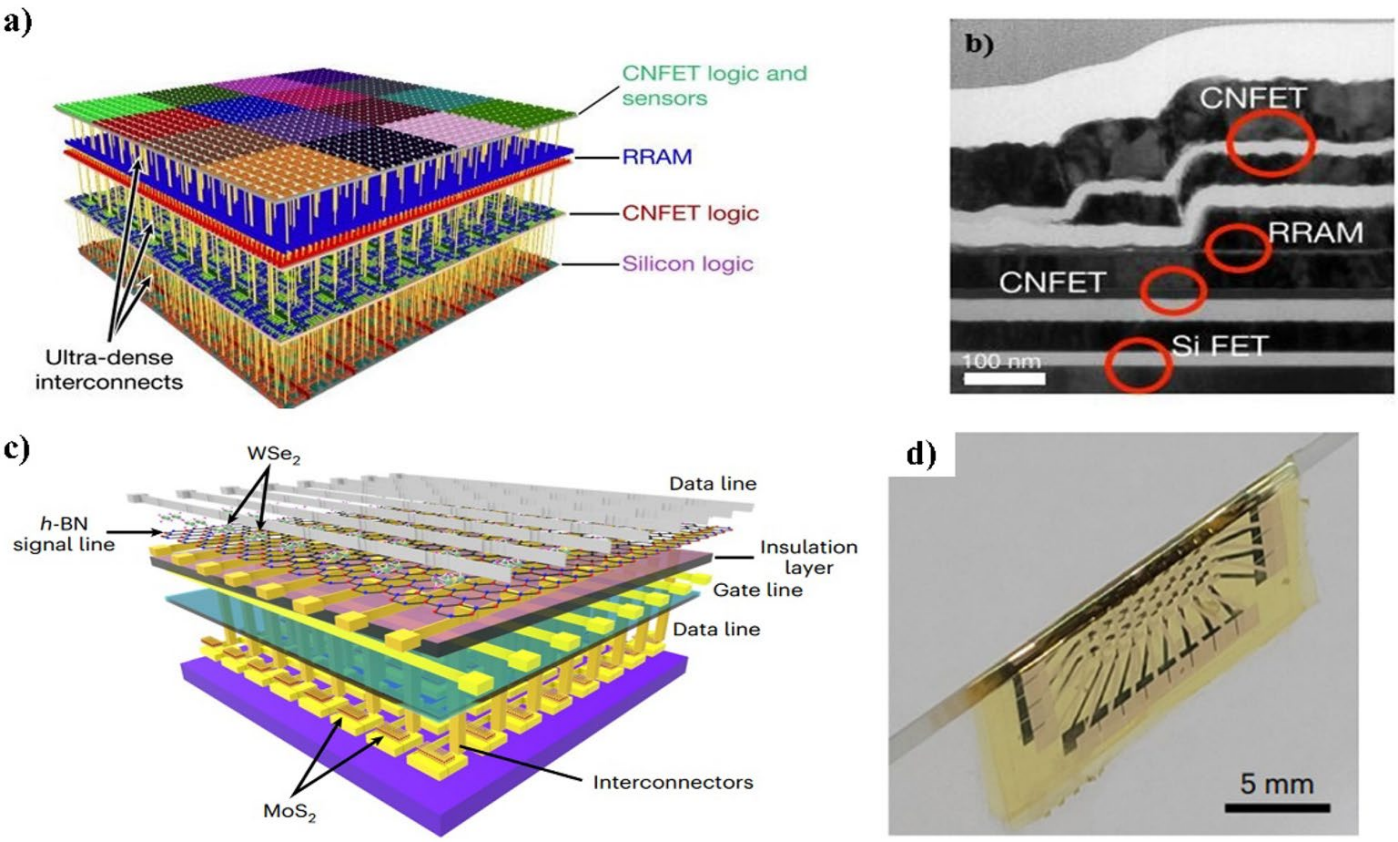
**a** Illustration and **b** cross-section TEM for carbon-nanotube FET in M3D structure [64] **c** Schematic diagram of M3D integrated with memristor and transistor layers **d** photograph of bendable AI processor [65]

2D materials, such as graphene and transition metal dichalcogenides (such as MoS_2_ and WS_2_) have attracted considerable attention because of their unique performances in extremely thin bodies, such as in-plane thermal conductivity and high mobility, are potential candidates for M3D integration. From the first concept of M3D with 2D materials by Kang et al. [63], there have been many efforts to build stacking 2D-based devices. The transfer technique can be used by growing a layer on a donor substrate at a high temperature (800−1000 ^o^C) and transferring the layer to the target substrates by tape or chemicals at low temperatures, such as carbon-nanotube field-effect transistors (FET) in Fig. 11a, b [64] for achieving a low-temperature deposition process for 2D material. Recently, another group achieved the M3D integration for artificial intelligence processing hardware by employing WSe_2_/h-BN memristor and MoS_2_ transistor layer [65]. The AI processing layers, synthesized from 2D materials using the bottom-up approach, are peeled and stacked to create a fully M3D integrated AI system as in Fig. 11c, d. The outstanding mechanical properties of this M3D-integrated AI device on a flexible substrate open possibilities for application in wearable AI platforms. The indirect growing technique, however, has the limitation of being nearly winkle-free and residual-free. On the other hand, 2D materials are also deposited directly by chemical vapor deposition or sputtering at temperatures lower than 500 ^o^C of deposition temperature, which is available for wafer-level uniformity [66, 67]. Developing a bottom-up approach to enable the area-selective growth of 2D layers on CMOS is necessary because of the challenge in the etching process. Another potential application of 2D materials in M3D integration is as a transition layer between different materials to prevent lattice mismatch and improve the overall device performance. For example, graphene can be used as a buffer layer between III−V compounds and Si substrates, reducing the lattice mismatch and improving the epitaxial layer quality [68].

In addition to the great performance of 2D materials, there are many channel property challenges to commercializing these devices, such as contact resistance, doping process, and interfaces. Using the semimetal-semiconductor bismuth, the contact resistance, which can be reduced to 123 Ω.µm by avoiding gap-state pining, is the best-reported result thus far [69]. The doping process normally leads to poor surface stability and is incompatible with processes used in the current semiconductor industry. The use of 2D materials in M3D integration also has difficulty achieving large-scale synthesis and transfer of high-quality 2D materials and integrating 2D materials with existing fabrication processes. Nevertheless, ongoing research shows promise for using 2D materials in M3D integration.

### Germanium and III-V compounds

Ge and III−V compounds are attractive candidates for top-layer device fabrication in the M3D process because of their excellent electrical properties and potential low processing temperature. For germanium, it has high electron and hole mobility (3900 and 1500 cm^2^V^-1^s^-1^ for electron mobility, 1900 and 450 cm^2^V^-1^s^-1^ for hole mobility), which can lead to faster and more efficient transistors. The smaller bandgap of Ge compared to Si (0.67 and 1.12 eV) allows Ge to be used in a wider range of applications, such as high-speed optoelectronics and energy harvesting devices. In addition, it also has a higher thermal conductivity (60 W/mK), which can help dissipate heat in 3D integrated circuits. In the case of III−V semiconductor compounds (such as GaAs and InP), they also have high electron mobilities for high-speed electronics. Ge and III-V compounds can be integrated into the M3D structure using expitaxial layer transfer.

**Fig. 12:**
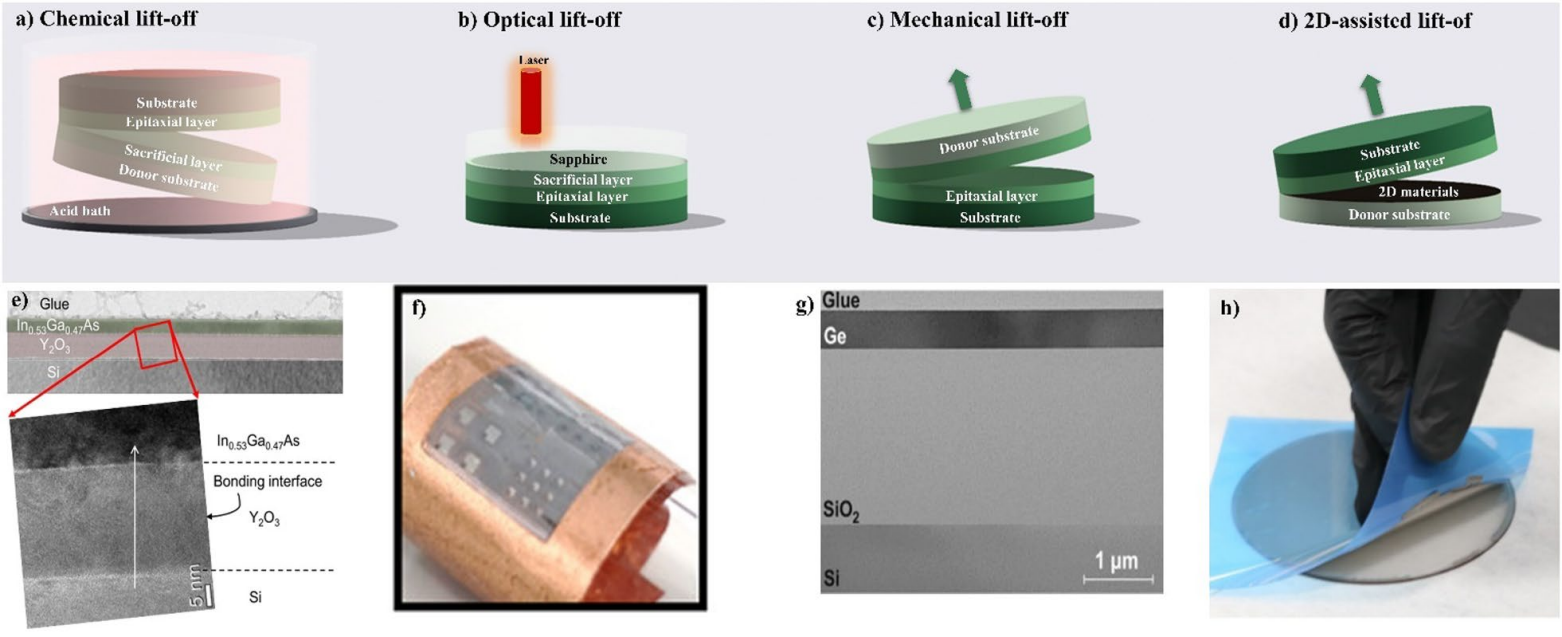
**a–d** Schematic of four types of epitaxial layer transfer **e** TEM image of In_0.53_Ga_0.47_As-on-insulator on a silicon substrate illustrates a highly uniform layer on Y_2_O_3_. The zoom in area shoes the excellent crystal quality with successful direct wafer bonding [70] **f** GaN LED chips is transferred successfully to Cu foil by laser lift-off process and integrated in an array [71] **g** High quality of Ge is observed after transfering by ion-cut process and CMP for reducing surface roughness [72] **h** thermal release tape was used for lift-off the stressed Ni layer on top of GaN, which provided the energy needed to break bondsat the boron nitride interface [73]

Epitaxial layer transfer techniques are gaining significance for creating thin, flexible, and 3D-integrated structures, offering two main advantages. Firstly, they enable the integration of dissimilar materials for expanded functionality, a feat challenging through conventional means. Secondly, these techniques allow for the reuse of the host substrates, significantly reducing fabrication costs. Various methods exist, including chemical lift-off, laser lift-off, mechanical lift-off, and 2D-assisted lift-off [74]. Chemical lift-off involves inserting a sacrificial layer that can be selectively etched as shown in Fig. 12a. For example, InGaAs is grown on an AlAs sacrificial layer, which is developed on the InP donor substrate. The InGaAs devices were patterned and bonded to the insulator substrate directly. The AlAs substrate was then removed by etching the sacrificial layer, leaving the InGaAs layer on the Si substrate as shown in Fig. 12e [70]. In this process, the interface quality, thermal mismatch, and limited scalability need to be noticed and studied.

Optical lift-off uses excimer lasers to separate epitaxial layers from transparent substrates as shown in Fig. 12b, allowing fast and robust separation but with limitations on material scope. Yulianto et al. used a laser with 520 nm for wavelength and 350 fs for pulse width for femtosecond laser lift-off. This process was conducted by scanning a laser beam across the backside of a sapphire substrate to separate and transfer processed GaN LED chips onto a target substrate (Cu foil) as shown in Fig. 12f [71]. The integrated fluence values between 2.5 and 4.5 J/cm^2^ were used for successful lift-off.

Mechanical lift-off, in which a buried layer of hydrogen ions is introduced into the substrate by ion implantation, followed by wafer bonding with another substrate, as shown in Fig. 12c, g. Ge on insulator (GOI) layers were fabricated by transferring a thin layer of single-crystal Ge onto a silicon substrate coated with a buried oxide layer [72, 75]. Hydrogen ion implantation produces a buried cavity in the germanium layer, which is then bonded to a handle wafer to form the GOI structure. The implanted ions can be activated by heat treatment, causing them to expand and cleave the top layer of the substrate, leaving a clean and smooth surface for subsequent processing. This technique, however, has some drawbacks, such as the degradation of device performance because of crystal damage defects and impurities, and the challenge of controlling and precise alignment for implantation parameters.

The 2D-assisted transfer technique combines benefits from van der Waals epitaxy, remote epitaxy, and 2D material-assisted transfer, offering controlled spalling depth and an atomically sharp separation interface as shown in Fig. 12d. In vdW epitaxy, epitaxial growth on 2D materials is facilitated by weak vdW interactions, allowing easy release of layers from 2D surfaces. The results of some researches proved that single-crystalline films as GaAs, InP, and GaP could be rapidly released from the graphene-coated substrate and perform well when incorporated into light-emitting devices [76]. In other result, boron nitride (BN) layer was also used as buffer layer for lift-of 4-inch GaN layey process as shown in Fig. 12h [73]. Characterizaton mapping reveals the excellent quality and uniformity of the GaN/AlN/BN stack grown in a single MOCVD run. The use of Ni spalling for lift-off, combined with a BN vdW release layer, demonstrates the scalability, speed, and yield of the process. Post-transfer characterization indicates a low impact on GaN quality while effectively relaxing significant residual strain formed during high-temperature growth. However, challenges remain, such as the inability to grow certain elemental semiconductors through remote epitaxy and the need for further development for true wafer-scale applications. Despite challenges, these techniques have demonstrated success at various scales, particularly in applications like thin-film solar cells and device layers on flexible substrates.

## Conclusion

Various channel techniques open a large window for the M3D process from concept to reality. Direct wafer bonding, ion-cut, and laser annealing are options for upper CMOS layers, while materials such as IGZO, 2D materials, Ge, and III−V compounds, are good choices for optical, sensor, or flexible devices. Substantial progress has been made in the development of upper active channel layers. Nevertheless, each technique faces distinct challenges, such as voids on the contact surface in wafer bonding, surface roughness in laser annealing, the subpar performance of IGZO, and the fabrication process for 2D materials. Addressing these issues is crucial for the successful commercialization of these devices.

## Data Availability

Not applicable.

## Abbreviations

2D: Two dimensions
3D: Three dimensions
M3D: Monolithic 3D
TVS: Through-via-silicon
poly-Si: Polycrystalline silicon
SCS: Single-crystal silicon
LPCVD: Low-pressure chemical vapor deposition
PECVD: Plasma-enhanced chemical vapor deposition
LA: Laser annealing
MIC: Metal-induced crystallization
MILC: Metal-induced lateral crystallization
FLA: Flash-lamp annealing
a-Si: Amorphous silicon
CW: Continuous wave
UV: Ultraviolet
DUV: Deep ultraviolet
CMOS: Complementary metal-oxide-semiconductor
BLDA: Blue-multi-laser-diode
TFT: Thin-film transistor
ILD: Interlayer dielectric
CMP: Chemical-mechanical polishing
FET: Field-effect transistor
GOI: Ge on insulator
